# Characterisation of the Wildlife Reservoir Community for Human and Animal Trypanosomiasis in the Luangwa Valley, Zambia

**DOI:** 10.1371/journal.pntd.0001211

**Published:** 2011-06-21

**Authors:** Neil E. Anderson, Joseph Mubanga, Eric M. Fevre, Kim Picozzi, Mark C. Eisler, Robert Thomas, Susan C. Welburn

**Affiliations:** 1 Centre for Infectious Diseases, Division of Pathway Medicine, College of Medicine and Veterinary Medicine, The University of Edinburgh, Edinburgh, United Kingdom; 2 Tsetse Control Section, Chilanga, Zambia; 3 Centre for Infectious Diseases, Institute of Immunology and Infection Research, Ashworth Laboratories, The University of Edinburgh, Edinburgh, United Kingdom; 4 Animals, Conservation and Education Department, The Royal Zoological Society of Scotland, Edinburgh Zoo, Edinburgh, United Kingdom; Foundation for Innovative New Diagnostics (FIND), Switzerland

## Abstract

**Background:**

Animal and human trypanosomiasis are constraints to both animal and human health in Sub-Saharan Africa, but there is little recent evidence as to how these parasites circulate in wild hosts in natural ecosystems. The Luangwa Valley in Zambia supports high densities of tsetse flies (*Glossina* species) and is recognised as an historical sleeping sickness focus. The objective of this study was to characterise the nature of the reservoir community for trypanosomiasis in the absence of influence from domesticated hosts.

**Methodology/Principal Findings:**

A cross-sectional survey of trypanosome prevalence in wildlife hosts was conducted in the Luangwa Valley from 2005 to 2007. Samples were collected from 418 animals and were examined for the presence of *Trypanosoma brucei* s.l., *T. b. rhodesiense*, *Trypanosoma congolense* and *Trypanosoma vivax* using molecular diagnostic techniques. The overall prevalence of infection in all species was 13.9% (95% confidence interval [CI]: 10.71–17.57%). Infection was significantly more likely to be detected in waterbuck (*Kobus ellipsiprymnus*) (Odds ratio [OR] = 10.5, 95% CI: 2.36–46.71), lion (*Panthera leo*) (OR = 5.3, 95% CI: 1.40–19.69), greater kudu (*Tragelaphus strepsiceros*) (OR = 4.7, 95% CI: 1.41–15.41) and bushbuck (*Tragelaphus scriptus*) (OR = 4.5, 95% CI: 1.51–13.56). Bushbucks are important hosts for *T. brucei* s.l. while the Bovidae appear the most important for *T. congolense*. The epidemiology of *T. vivax* was less clear, but parasites were detected most frequently in waterbuck. Human infective *T. b. rhodesiense* were identified for the first time in African buffalo (*Syncerus caffer*) and *T. brucei* s.l. in leopard (*Panthera pardus*). Variation in infection rates was demonstrated at species level rather than at family or sub-family level. A number of significant risk factors interact to influence infection rates in wildlife including taxonomy, habitat and blood meal preference.

**Conclusion and Significance:**

*Trypanosoma* parasites circulate within a wide and diverse host community in this bio-diverse ecosystem. Consistent land use patterns over the last century have resulted in epidemiological stability, but this may be threatened by the recent influx of people and domesticated livestock into the mid-Luangwa Valley.

## Author Summary

Animal and human trypanosomiasis are constraints to both animal and human health in Sub-Saharan Africa, but there is little recent evidence as to how these parasites circulate in natural hosts in natural ecosystems. A cross-sectional survey of trypanosome prevalence in 418 wildlife hosts was conducted in the Luangwa Valley, Zambia, from 2005 to 2007. The overall prevalence in all species was 13.9%. Infection was significantly more likely to be detected in waterbuck, lion, greater kudu and bushbuck, with a clear pattern apparent of the most important hosts for each trypanosome species. Human infective *Trypanosoma brucei rhodesiense* parasites were identified for the first time in African buffalo and *T. brucei* s.l. in leopard. Variation in infection is demonstrated at species level rather than at family or sub-family level. A number of significant risk factors are shown to interact to influence infection rates in wildlife including taxonomy, habitat and blood meal preference. *Trypanosoma* parasites circulate within a wide and diverse host community in this bio-diverse ecosystem. Consistent land use patterns over the last century have resulted in epidemiological stability, but this may be threatened by the recent influx of people and domesticated livestock into the mid-Luangwa Valley.

## Introduction

Trypanosomes are true multi-host parasites capable of infecting a wide range of wildlife species that constitute a reservoir of infection for both people and domestic animals. Natural infections of trypanosomes in wildlife were first identified during the Sleeping Sickness Commission in the Luangwa Valley, Zambia [1], which had been set up following the identification of the first case of the ‘rhodesian’ form of human sleeping sickness in 1910 [2]. Since that time, many surveys from across Africa have identified extensive natural infection of wild animal hosts with a variety of trypanosome species [3]–[18]. Although much knowledge about infection rates in wildlife has been generated, there have been very few attempts to conduct epidemiological analyses of data from surveys of wildlife. This is, in part due to difficulties in the collection of sufficiently large sample sizes of a representative nature from wildlife species to enable a thorough statistical analysis [19]–[21]. Some studies have examined the association between wildlife infection rates and factors that might influence them [8], [17], but most have reported host species infection rates without any statistical analysis of the data. Consequently, knowledge of the factors associated with trypanosome infection in wildlife is limited.

In the Luangwa Valley, high tsetse (and trypanosome) challenge coupled with high levels of predation and an arid environment, have meant that livestock keeping has been virtually non-existent in this area. The majority of the land is protected for the conservation of the environment and industries based around both consumptive and non-consumptive tourism form the main sources of revenue for local peoples. Pressure for land is lower than in many wildlife areas of Africa, although there has been an influx of people from Eastern Province into the Central Luangwa Valley in recent years. Three general surveys of trypanosome infections of wildlife have been conducted [1], [7], [13]. All three surveys have utilised parasitological methods of diagnosis, although the study by Keymer, 1969 combined parasitological identification with rodent inoculation and used the blood incubation infectivity test (BIIT) [22] to investigate the presence of human infective *T. b. rhodesiense*. This human infective, zoonotic subspecies has been identified in bushbuck (*Tragelaphus scriptus)*, duiker (*Sylvicapra grimmia*), giraffe (*Giraffa camelopardalis thornicrofti*), impala (*Aepyceros melampus*), lion (*Panthera leo*), warthog (*Phacocoerus africanus*) and waterbuck (*Kobus ellipsiprymnus*) [23], impala and zebra (*Equus quagga boehmi*) [24] and warthog [7], [25] in the Luangwa Valley to date.

The investigation of the wildlife reservoir for the human infective subspecies has for a long time been complicated by difficulties in the definitive diagnosis of the parasite. Although the BIIT [22] represented a significant advance in diagnostic capabilities, recent advances in molecular diagnostic methods have resulted in the development of a new, highly specific and robust diagnostic tests for this parasite [26]. Advances in molecular methods of diagnosis have also resulted in the development of a multispecies polymerase chain reaction (PCR) that is capable of differentiating all the major pathogenic trypanosomes of domestic livestock in a single test [27]. This study aimed to apply these novel laboratory techniques to further characterise the wildlife reservoir for trypanosomes in the Luangwa valley. A statistical analysis of infection rates in wildlife was to be used to identify the principle components of the wildlife reservoir community for *T. brucei* s.l., *T. congolense* and *T. vivax*, in the absence of any domesticated animal hosts. Additionally, in order to understand the transmission of trypanosomes better, the data was to be used to identify risk factors for infection.

## Methods

### Study design

A cross-sectional survey of the trypanosomiasis prevalence in wildlife hosts was conducted in the Luangwa Valley from 2005 to 2007. The Luangwa Valley is situated in the Eastern and Northern provinces of Zambia and represents an extension of the Great Rift Valley in East Africa. Sample collection took place between May and November of each year as the main roads north and south within the Luangwa Valley are impassable during the rainy season. Due to the inherent difficulty in collecting samples from wildlife in remote areas samples were collected using non-randomised convenience sampling techniques.

**Figure 1:**
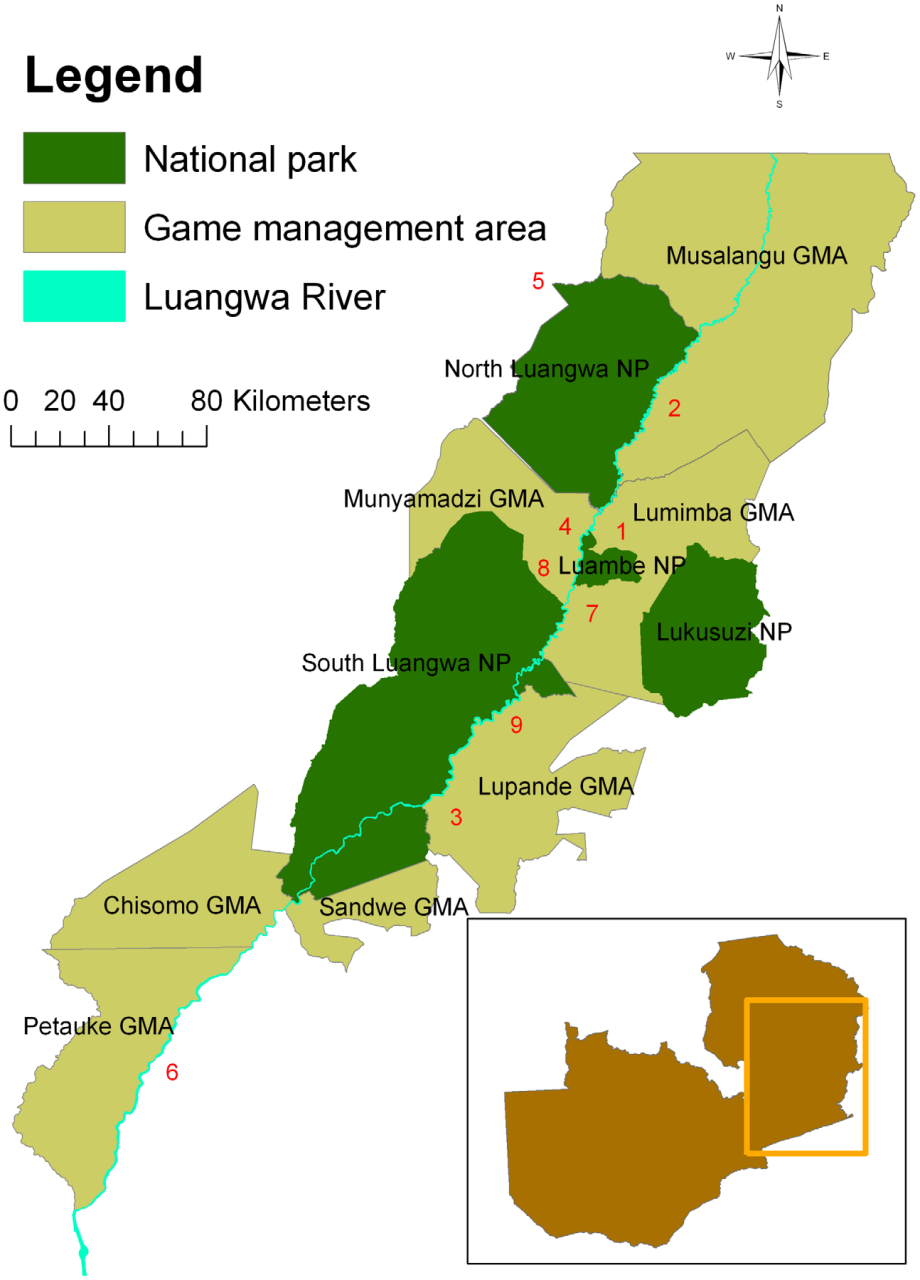
Study area showing the national parks and game management areas. Hunting blocks: 1 - Chanjuzi / Nyaminga; 2 - Chifunda; 3 - Lower Lupande; 4 Luawata; 5 - Mukungule; 6 - Munyamadzi Game Ranch; 7 - Mwanya; 8 - Nyampala; 9 - Upper Lupande. The inset figure shows the approximate location of the Luangwa Valley in Zambia.

Samples were collected using two sampling approaches. Firstly, professional hunters were recruited to provide samples from animals harvested as part of the commercial ‘safari hunting’ system operating in Zambia. These animals are shot under licence in game management areas (GMAs) outside the national parks and hunting licenses are issued under a quota system regulated by the Zambian Wildlife Authority (ZAWA). The study area covered eight GMAs with a widespread distribution across the north and central Luangwa Valley and one private hunting area further south in Luembe Chiefdom (Figure 1). Additional samples were also collected from animals destroyed in GMAs as part of population control measures or problem animal control.

Secondly, samples were collected from animals immobilised or captured as part of routine conservation management activities within the national parks of the Luangwa Valley. The majority of the animals sampled were captured in North Luangwa National Park (NLNP) by a commercial game capture unit as part of a re-stocking programme for the Malawi / Zambia Transfrontier Conservation Area. Additional samples were collected from South Luangwa National Park and Lower Lupande GMA. The overall study area covered represents the largest area covered by any trypanosomiasis survey of wildlife in the Luangwa Valley to date.

### Ethics statement

This study utilised blood samples collected from wild animals that had already been shot as part of commercial safari hunting activities under a strictly licensed quota system managed by the Zambian Wildlife Authority. These animals were not shot for the purpose of this study. Additional samples were also collected from animals captured and released unharmed as part of a translocation exercise for the Zambia / Malawi Transfrontier Conservation Area. All activities in protected areas were fully approved by the Zambian Wildlife Authority (permit numbers 316295 and 323947). All sampling protocols were approved by the Zambian Wildlife Authority and the Zambian Department of Veterinary and Livestock Development. The relevant export and import licences were obtained for samples from animals on CITES appendices 1 and 2.

### Sample collection and storage

Blood samples were collected onto FTA® Cards (Whatman, Maidstone, Kent, UK) and were left to air dry out of direct sunlight. Samples were stored in multi-barrier pouches (Whatman) with desiccant at ambient temperature prior to being processed in the laboratory. Samples collected by hunters were collected either directly from the bullet wound immediately after the animal was shot, or at the time of skinning from heart blood or muscle smeared onto the FTA® card. A small number of samples (26) were collected onto Isocode cards (Schleicher & Schuell, Dassel, Germany), rather than FTA® cards, and were stored in a similar manner. The remaining samples were collected from a superficial ear vein into heparinised capillary tubes and applied to FTA® Cards. Where sufficient amounts of blood could not be obtained from an ear vein, blood samples were collected from larger peripheral veins (jugular, saphenous, cephalic or abdominal) into EDTA coagulated vacutainers and stored at 4° centigrade prior to application to FTA® Cards.

Each sample was assigned a unique identification number. Supplementary data on the species, sex, age, date and location were recorded. Age was recorded using knowledge of breeding seasons and examination of the physical maturity of the animal to assign it to one of three categories: (i) born in the present breeding season or within the last six months (juvenile); (ii) born the previous breeding season, but not yet mature (sub-adult); (iii) physically mature (adult). For the samples collected through the professional hunter survey, Global Positioning System (GPS) coordinates were requested for all samples, but a number of hunters were unable to provide this information. GPS coordinates were recorded for all other samples using a hand-held Garmin GPS device.

### Sample preparation

All samples collected were screened using PCR methods. Eluted deoxyribonucleic acid (DNA) was used to seed each PCR and was prepared using the following sample preparation protocols. Samples collected onto Whatman FTA® Cards were firstly prepared for elution by punching five 3 mm diameter discs from each card using a Harris Micro-Punch ™ Tool. The use of multiple sample punches from each card increases the likelihood of detection of trypanosomes present at low levels of parasitaemia [28]. The discs were then washed twice in 1000 µl of Whatman Purification Reagent for 15 minutes followed by two washes in 1000 µl of 1× concentrated TE buffer for 15 minutes to remove any residual Whatman purification reagent. The discs were then transferred to 100 µl PCR tubes, with all five discs from each sample placed in one tube, and allowed to dry at room temperature. Once the discs had dried, a Chelex 100® elution protocol was used to elute the DNA [29]. 100 µl of 5% (w/v) Chelex solution (sodium form, 100–200 mesh; Bio-Rad Laboratories, Hemel Hempstead, Hertfordshire, UK) was added to each sample and mixed thoroughly by pipetting. The samples were then heated to 90°C for 30 minutes in a DNA Engine DYAD™ Peltier Thermal Cycler. Eluted DNA was stored at 4°C and used in a PCR within 12 hours of elution. Unused sample DNA was stored in aliquots at −20°C for longer periods.

Samples collected onto Isocode cards, were prepared for elution by punching three 3 mm diameter discs from each sample card and placing them together in a sterile 1.5 ml microcentrifuge tube. 500 µl of sterile de-ionised water (dH_2_O) was added and the tubes pulse vortexed for five seconds a total of three times. The discs were gently squeezed against the side of the tube and placed in a sterile 0.5 ml microcentrifuge tube. The DNA was eluted using a simple water elution protocol. 100 µl of dH_2_O was added and the tubes were then heated to 95°C for thirty minutes in a DNA Engine DYAD™ Peltier Thermal Cycler. The tubes were gently tapped twenty times to mix the sample and the discs containing the matrix were removed. The eluate was stored at 4°C and used in a PCR within 12 hours of elution. Excess sample eluate was stored at −20°C in 10 µl aliquots.

### Molecular analysis

Samples were initially screened using a multispecies nested PCR that distinguishes all clinically important African trypanosome species and some sub-species [27]. The PCR targets the Internal Transcribed Spacers (ITS) of the small ribosomal subunit (200 copies per genome), producing different sized products for different trypanosome species. ITS-PCR was performed on each sample in a standard reaction volume of 25 µl using 1 µl of eluate as a template for each reaction, under the reaction conditions described by Cox *et al* [27].

All samples were also screened using a species-specific PCR for *T. brucei* s.l. [30]. The TBR-PCR is a species specific PCR for trypanosomes belonging to the *Trypanozoon* subgenus. The primers are designed to amplify a target region with a copy number of 10,000 per genome making it a highly sensitive test. The PCR was carried out on each sample using 5 µl of eluate in a standard reaction volume of 25 µl under the reaction conditions described by Moser *et al* [30].

Any samples detected as being positive for *T. brucei* s.l. using either of the above PCRs were then subjected to a multiplex PCR for the detection of the *T. brucei rhodesiense* subspecies [26], [31]. The two *T. brucei* s.l. subspecies, *T. brucei brucei* and *T. brucei rhodesiense* are distinguished by the presence of the SRA gene in the latter's genome. The multiplex PCR is designed to amplify the SRA gene, thereby enabling the differentiation of the two subspecies. As the SRA gene is a single copy gene, primers amplifying the single copy GPI-PLC gene are also included within the PCR as a positive control to show that enough genomic material was present for the SRA gene to be detected if present. A failure to detect the GPI-PLC gene in TBR-PCR positive samples might suggest that the prevalence of *T. b. rhodesiense* was being underestimated. 5 µl of eluate from each positive sample was used in a standard reaction volume of 25 µl under the reaction conditions described by Picozzi *et al* [26]. In order to improve the sensitivity of the method, the reaction was run three times for each sample using 45 cycles and three times using 50 cycles.

In all PCRs, a positive control (genomic DNA) and two negative controls (blank FTA punch and water) were run with each reaction. A DNA Engine DYAD™ Peltier Thermal Cycler was used to run the reactions and PCR products were separated by electrophoresis in a 1.5% (w/v) agarose gel containing 0.5 µg/ml ethidium bromide. Separated products were then visualised under ultraviolet light in a transilluminator.

### Statistical analysis

Logistic regression models with binomial errors were used for the investigation of trypanosomiasis prevalence. Data was initially entered and evaluated using the Microsoft ®Office Excel 2003 spreadsheet program. All analytical exploration of data was conducted using the statistical software package, R: A language and environment for statistical computing [32]. Additional functions included within the Epicalc 2.7.1.2. package for R (V. Chongsuvivatwong) were also used in the analysis. The likelihood ratio test was used to assess the significance of individual factors in each model. For individual factor categories, the likelihood of infection in comparison to the reference category was presented as the odds ratio (OR). The Walds statistic was used to assess the significance of the OR and is presented as the probability (p) value. Statistical significance was accepted at the 95% confidence level throughout the analysis.

The models were firstly used to investigate the overall prevalence detected of all trypanosome species combined. The analysis was then repeated to investigate the prevalence of *T. brucei* s.l., *T. congolense* and *T. vivax* separately. The effect of host species, age, sex, area, month and year on trypanosome prevalence detected was examined using each factor as an explanatory variable. The potential confounding effect of sample collection method was also investigated with each sample being assigned to a category according to whether it was sampled alive or dead. The data from the samples collected by hunters were also initially analysed as a separate dataset as were the data from the samples collected from national parks before the whole dataset was analysed together. Over-saturation of some FTA cards with blood was observed during laboratory analysis and there was a concern that excess haem might interfere with the PCR for these samples. To investigate this each sample was assigned to a non-oversaturated category if the colour of the eluate was clear and to an over-saturated category if the eluate was discoloured by residual blood pigments.

To further investigate the variation in prevalence between species and to facilitate a multivariable analysis, three methods of grouping the species sampled were compared. Firstly, a grouping based on the taxonomic classification of species at the sub-family level (or family level where no sub-family exists) was used. This followed the standard text by Wilson and Reeder [33]. Secondly, a grouping based on both the predominant vegetation type that the species favoured (open, closed or mixed) and the territorial or spatial movement patterns of the species (sedentary or non-sedentary) was investigated, again using the above text as a reference. Habitat was classified in this way in order to reflect potential association with preferred tsetse habitat. The final grouping method investigated was designed to reflect the blood meal preferences of the three tsetse species found in the Luangwa Valley. Clausen *et al's* publication on blood meal preferences [34] was used as reference to assign each wild animal species sampled from into one of three levels (low, medium and high) depending on the proportion of blood meals it accounted for. Although this publication contained many samples from Zambia, blood meal preferences were presented only by tsetse species not by geographical region so no regional values could be used. Additionally, many species sampled from were not included in the publication and for these species more general publications on host preferences were used to subjectively assign categories [35], [36]. The groupings and how they were calculated are summarised in Table 1. The data was initially explored at the univariable level and then at the multivariable level. However, multivariable analysis was only possible for overall trypanosome prevalence as there was inadequate data for the individual trypanosome species.

**Table 1:** Comparison of the methods used to group species for the analysis.

Species	Taxonomy group	Habitat group	Blood meal group
African painted dog	Canidae	Non-sedentary mixed	Low
Buffalo	Bovinae	Non-sedentary mixed	Medium
Bushbuck	Bovinae	Sedentary closed	High
Crocodile	Crocodilinae	Aquatic	Low
Duiker	Cephalophinae	Sedentary closed	Low
Eland	Bovinae	Non-sedentary closed	Medium
Elephant	Elephantidae	Non-sedentary mixed	Medium
Giraffe	Giraffidae	Non-sedentary closed	Medium
Greater Kudu	Bovinae	Sedentary closed	High
Grysbok	Antilopinae	Sedentary closed	Low
Hartebeest	Alcelaphinae	Sedentary open	Low
Hippo	Hippopotamidae	Aquatic	High
Hyaena	Hyaenidae	Sedentary closed	Low
Impala	Aepycerotinae	Sedentary mixed	Low
Leopard	Pantherinae	Sedentary closed	Low
Lion	Pantherinae	Non-sedentary mixed	Low
Puku	Reduncinae	Sedentary open	Low
Reedbuck	Reduncinae	Sedentary open	Low
Roan	Hippotraginae	Sedentary mixed	Low
Vervet monkey	Cercopithecinae	Sedentary closed	Low
Warthog	Suidae	Sedentary mixed	High
Waterbuck	Reduncinae	Sedentary closed	Low
Wildebeest	Alcelaphinae	Non-sedentary open	Medium
Zebra	Equidae	Non-sedentary open	Low

For blood meal group low = <5% of total blood meals, medium = 5–10%, high = >10%.

## Results

### Samples collected

In total 418 samples were collected from 24 species in the survey (Table 2). The majority of these were collected from GMAs through the professional hunter survey in which 331 samples were collected from 22 species. All of these samples were from adult animals and only four were from female animals. A total of 80 samples from five species were collected from the NLNP and these were more representative with 34 samples from male animals and 46 from females. Twenty-four sub-adults were sampled along with two juveniles, the remainder being adults. An additional seven samples were collected from the other management activities.

**Table 2:** Summary of the species and age distribution of animals sampled.

Species	Adult		Sub-adult		Juvenile		Total
	Male	Female	Male	Female	Male	Female	
African painted dog	1	1	1	0	0	0	3
Buffalo	63	1	0	0	0	0	65†
Bushbuck	27	1	0	0	0	0	28
Crocodile	4	1	0	0	0	0	5
Duiker	2	0	0	0	0	0	2
Eland	1	0	0	0	0	0	1
Elephant	2	4	0	0	0	0	7†
Giraffe	0	0	0	0	0	0	1†
Grysbok	4	0	0	0	0	0	4
Hartebeest	4	0	0	0	0	0	4
Hippo	15	0	0	0	0	0	29†
Hyaena	4	1	0	0	0	0	7†
Impala	42	0	4	0	0	0	47†
Kudu	20	0	0	0	0	0	20
Leopard	14	0	0	0	0	0	14
Lion	13	0	0	0	0	0	14†
Puku	40	10	4	2	0	0	57†
Reedbuck	1	0	0	0	0	0	1
Roan	5	0	0	0	0	0	5
Vervet monkey	1	0	0	0	0	0	1
Warthog	27	15	7	7	0	0	56
Waterbuck	10	0	0	0	0	0	10
Wildebeest	10	0	0	0	0	0	10
Zebra	14	9	2	0	0	2	27
Age total by sex	324	43	18	9	0	2	396
Age totals	367		27		2		396
Sex totals	Male = 342			Female = 54			396
Overall Total	418						

**†:** The age and sex of 14 hippo was unknown, the sex of 1 buffalo, 1 elephant, 1 giraffe, 2 hyaena, 1 impala, 1 lion and 1 puku was unknown.

### Overall trypanosome prevalence

The cumulative prevalence of all trypanosomes in the dataset was 13.9% (95% CI: 10.71–17.57%). Four mixed infections were detected giving an overall prevalence of mixed infections of 1.0% (95% CI: 0.26–2.43%). The percentage of total infections present as mixed infections was 6.9%. All involved *T. brucei* s.l., with three occurring concurrently with *T. congolense* (one each in a bushbuck, warthog and wildebeest (*Connochaetes taurinus cooksoni*)) and one occurring concurrently with *T. vivax* (in a waterbuck).

**Figure 2:**
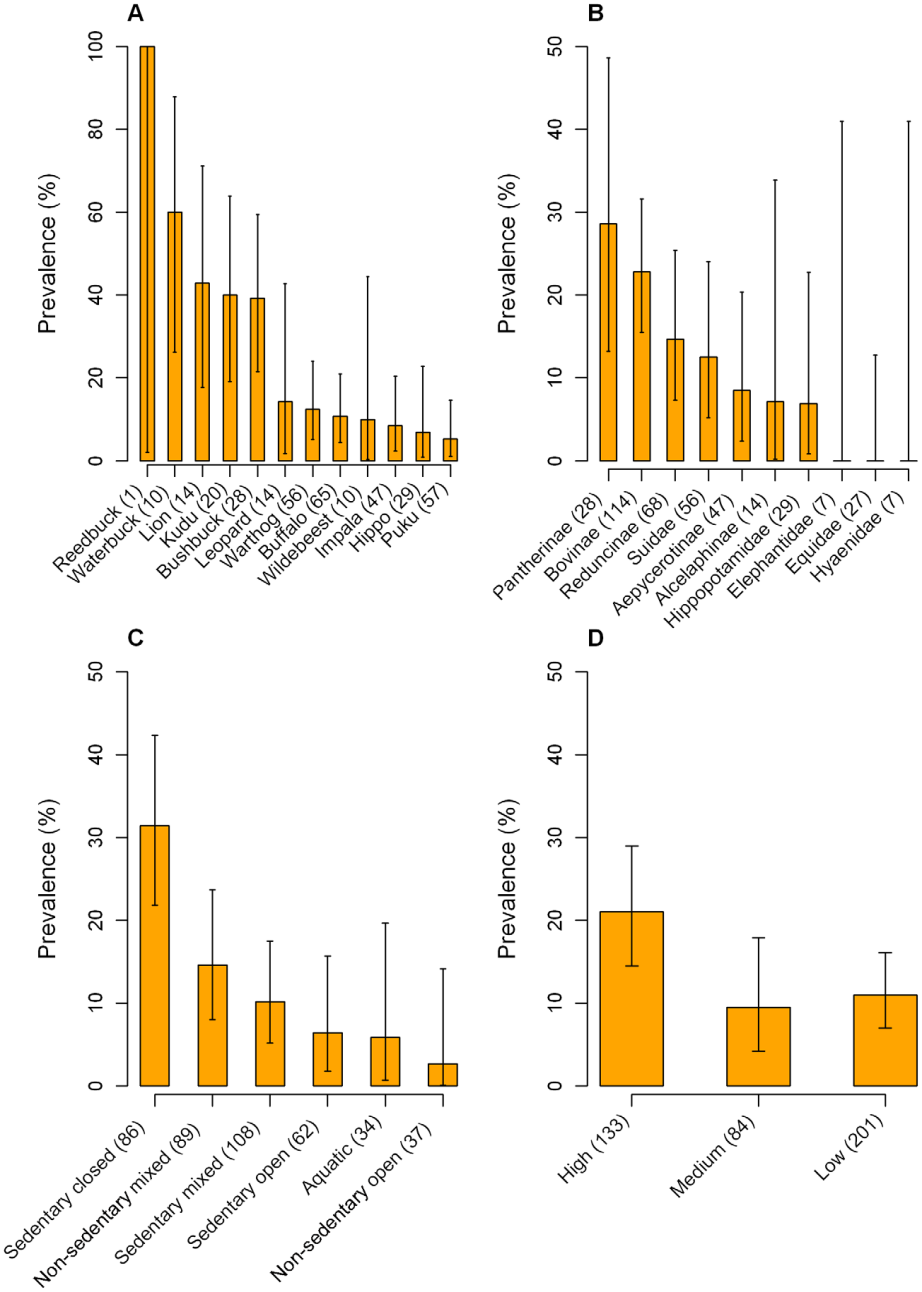
Bar charts of overall trypanosome prevalence. Prevalence is plotted against (A) wildlife species, (B) taxonomy group, (C) habitat group and (D) blood meal preference group. Only the species with at least one positive sample are shown in each graph. Error bars represent exact binomial 95% confidence intervals.

The effect of wild animal species on the overall prevalence of trypanosome infections was highly significant (p<0.001) and several species had a statistically significant increased risk of being infected with trypanosomes (Table 3). Waterbuck were the most likely species to be detected as being infected, with a significant OR of 10.5 (95% CI: 2.36–46.71, p = 0.002). Lion, greater kudu (*Tragelaphus strepsiceros*) and bushbuck were also significantly more likely to be detected as being infected, with respective ORs of 5.3 (95% CI: 1.40–19.69, p = 0.014), 4.7 (95% CI: 1.41–15.41, p = 0.012) and 4.5 (95% CI: 1.51–13.56, p = 0.007). The prevalence detected in each species with at least one positive sample is shown in Figure 2 (A).

**Table 3:** Summary of the univariable prevalence and odds ratios for overall trypanosome prevalence in each host species and taxonomy group.

Species (n)	Prevalence %(95% CI)	OR(95% CI)	Species group	Prevalence %(95% CI)	OR(95% CI)
Warthog (56)	12.50 (5.18–24.07)	Referent	Suidae	12.50 (5.18–24.07)	Referent
African paintedDog (3)	0 (0.00–71.00)	N/A	Canidae	-	-
Buffalo (65)	10.77 (4.44–20.94)	0.84 (0.28–2.58)	Bovinae	22.81 (15.47–31.61)	2.07 (0.84–5.11)
Bushbuck (28)	39.29 (21.50–59.42)	4.53 (1.51–13.56)**			
Eland (1)	0 (0.00–98.00)	N/A			
Greater kudu (20)	40.00 (19.12–63.95)	4.67 (1.41–15.41)*			
Crocodile (5)	0 (0.00–52.20)	N/A	Crocodilidae	-	-
Duiker (2)	0 (0.00–84.50)	N/A	Cephalophinae	-	-
Elephant (7)	0 (0.00–41.00)	N/A	Elephantidae	0 (0.00–41.00)	N/A
Giraffe (1)	0 (0.00–98.00)	N/A	Giraffidae	-	-
Grysbok (4)	0 (0.00–60.25)	N/A	Antilopinae	-	-
Hartebeest (4)	0 (0.00–60.25)	N/A	Alcelaphinae	7.14 (0.18–33.87)	0.54 (0.06–4.78)
Wildebeest (10)	10.00 (0.25–44.50)	0.78 (0.09–7.11)			
Hippo (29)	6.90 (0.85–22.77)	0.52 (0.10–2.67)	Hippopotimidae	6.90 (0.85–22.77)	0.52 (0.10–2.67)
Hyaena (7)	0 (0–41.00)	N/A	Hyaenidae	0 (0–41.00)	N/A
Impala (47)	8.51 (2.37–20.38)	0.65 (0.18–2.38)	Aepycerotinae	8.51 (2.37–20.38)	0.65 (0.18–2.38)
Leopard (14)	14.29 (1.78–42.81)	1.17 (0.21–6.35)	Pantherinae	28.57 (13.22–48.67)	2.80 (0.9–8.75).
Lion (14)	42.86 (17.66–71.14)	5.25 (1.40–19.69)*			
Puku (57)	5.26 (1.10–14.62)	0.39 (0.10–1.59)	Reduncinae	14.71 (7.28–25.39)	1.21 (0.43–3.41)
Reedbuck (1)	100.00 (2.00–100.00)	N/A			
Waterbuck (10)	60.00 (26.24–87.85)	10.5 (2.36–46.71)**			
Roan (5)	0 (0.00–52.20)	N/A	Hippotraginae	-	-
Vervet monkey (1)	0 (0.00–98.00)	N/A		-	-
Zebra (27)	0 (0.00–12.78)	N/A	Equidae	0 (0.00–12.78	N/A

N/A – Odds ratios could not be calculated due to zero prevalence or small sample size.

**p<0.01;

*p<0.05; . p<0.1.

The effect of taxonomy group on the overall trypanosome prevalence was highly significant (p = 0.002). However, no individual taxonomy group had a significantly increased risk of being infected compared with the reference Suidae group. The group with the highest prevalence was the Pantherinae and the odds of this group being detected as infected with trypanosomes approached significance (OR = 2.8, 95% CI: 0.90–8.75, p = 0.077). The prevalence detected in each sub-family is shown in Figure 2 (B). The effect of habitat group was also highly significant as a factor (p<0.001) with the sedentary closed habitat group significantly more likely to be infected than the reference sedentary open habitat group (OR = 6.6, 95% CI: 2.18–20.15, p<0.001) (Figure 2 (C)). Both sedentary and non-sedentary mixed habitat groups had an increased likelihood of being infected, but this was not significant in either group. Using Tukey contrasts as a method of the multiple comparison of means, the sedentary closed habitat group had a significantly higher prevalence of trypanosome infections than both the sedentary mixed and sedentary open groups (p = 0.004 and p = 0.009, respectively).

The effect of blood meal preference group was also significant as a factor (p = 0.018). The high blood meal preference group had an increased risk of being infected (p = 0.013) with an OR of 2.2 (95% CI: 1.18–3.99) compared to the reference low blood meal preference group. Interestingly, the likelihood of the medium blood meal preference group being infected was lower than the reference group (OR = 0.9, 95% CI: 0.37–2.01, p = 0.722), but this difference was not statistically significant. The prevalence detected by blood meal preference group is shown in Figure 2 (D).

**Figure 3:**
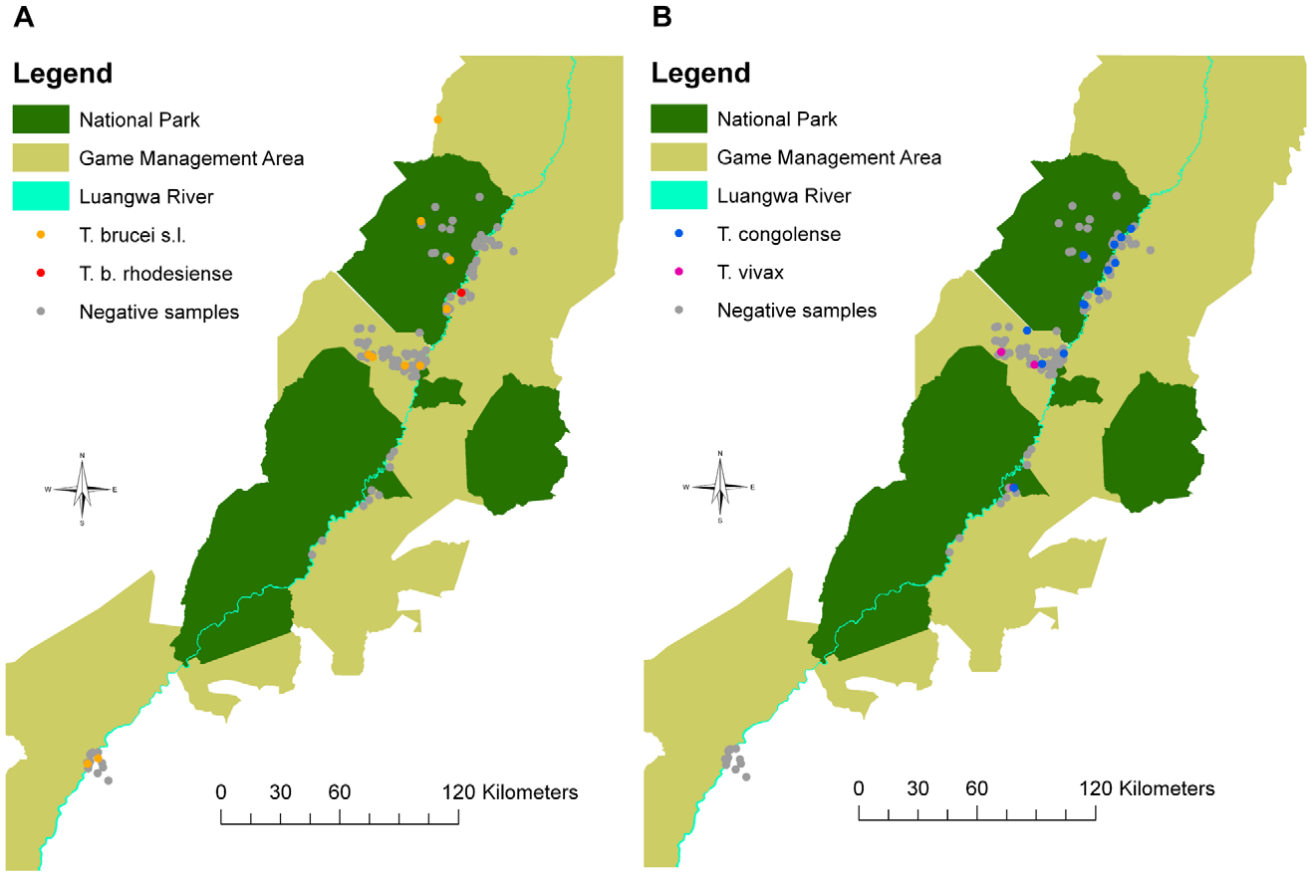
Spatial distribution and infection status of samples collected. (A) *T. brucei* s.l. and *T. b. rhodesiense*, (B) *T. congolense* and *T. vivax*. GPS data were lacking for many of the samples collected from professional hunters and these samples are not represented in the figures.

There were no significant effects of age on the prevalence of trypanosomes detected in any of the datasets. Sex was found to be a significant factor (p = 0.028), with male animals having higher odds of being infected than females (OR = 3.2, 95% CI: 0.96–10.58, p = 0.058). However, this effect was confounded by species as only one female sample was collected from a species with a high prevalence of trypanosomes. When adjusted for species the effect was no longer significant (p = 0.544) and the adjusted OR was lower (OR = 1.5, 95% CI: 0.38–6.15, p = 0.554). There were no significant effects of area on the prevalence of trypanosome infection and no spatial patterns were apparent in the data (Figure 3). No significant effects of the year of sampling were detected. Although month of sampling had a significant effect (p = 0.028), most of the samples from NLNP were collected in September and the species sampled had a lower prevalence. When the effects of month were adjusted for confounding by area the effect was no longer statistically significant (p = 0.248). Although sample collection method appeared to have a significant effect (p = 0.002), this was no longer the case when adjusted for either species (p = 0.267) or area (p = 0.432). The effect of over-saturating Whatman FTA or Isocode cards with blood was not statistically significant when the whole dataset was considered. However, when the samples collected by hunters were considered alone the effect approached statistical significance (p = 0.059) and samples that were classified as being over-saturated had a reduced likelihood of being detected as infected (OR = 0.6, 95% CI: 0.31–1.03, p = 0.063). When combined with the rest of the data, the effect became statistically insignificant (p = 0.209), but the odds of being detected as infected was still lower for over-saturated samples (OR = 0.7, 95% CI: 0.39–1.24, p = 0.215). The results of the univariable analysis of risk factors for overall infection with trypanosomes are summarised in Table 4.

**Table 4:** Univariable Analysis of deviance table for infection with all trypanosome species using the combined dataset.

*Grouping*	*DF*	*Deviance*	*Residual DF*	*Residual Deviance*	*LRT P value*
Species	23	69.61	394	267.04	<0.001
Taxonomy	9	26.17	387	304.03	0.002
Habitat	5	29.85	410	306.20	<0.001
Blood meal preference	2	8.09	415	328.57	0.018
Age	1	1.60	401	326.9	0.21
Sex	1	4.84	394	321.51	0.03†
Area	11	18.89	397	315.04	0.06
Year	2	0.08	415	336.58	0.96
Month	6	14.16	400	315.55	0.03†
Sample collection method	1	10.04	406	323.59	0.002†
Saturation status	1	1.58	415	334.78	0.21

**†:** No longer significant when adjusted for confounding.

A multivariable analysis was conducted using the taxonomy grouping of species. This grouping method was selected for the final analysis as it had the lowest residual deviance. However, despite the grouping of species, the nature of the data resulted in large standard errors so a reduced dataset (326 observations) with all species with a sample size less than five or no positive samples removed was used. The final multivariable model included taxonomy grouping and over-saturation of sample cards, with area included as a confounding variable. No factors were significant, but the effect of over-saturation approached significance (p = 0.054) with over-saturated cards less likely to be detected as infected (OR = 0.5, 95% CI: 0.28–1.02, p = 0.058). Infection rates were highest in Pantherinae (OR = 2.0, 95% CI: 0.53–7.27), Bovinae (OR = 1.6, 95% CI: 0.57–4.59) and Reduncinae (OR = 1.2, 95% CI: 0.41–3.71) taxonomy groups.

### Prevalence of *T. brucei* species

The overall cumulative prevalence of *T. brucei* s.l. in all species was 5.7% (95% CI: 3.71–8.42%). The prevalence detected using the individual species PCR for *T. brucei* s.l. was 5.3% (95% CI: 3.33–7.86%) compared with 0.5% (95% CI: 0.06–1.72%) using the multispecies PCR. Two *T. b. rhodesiense* infections were detected using the SRA-PCR giving a prevalence of 0.5% (95% CI: 0.06–1.72%). The positive samples came from a male adult bushbuck from Chifunda hunting block in Musalangu GMA and a male adult buffalo (*Syncerus caffer*) from the Nyamaluma area of Lower Lupande GMA. The proportion of all *T. brucei* s.l. infections that were identified as *T. b. rhodesiense* was therefore 0.08, or 8.3%. However, the GPI-PLC gene was not detected in the majority of the *T. brucei* s.l. positive samples.

**Figure 4:**
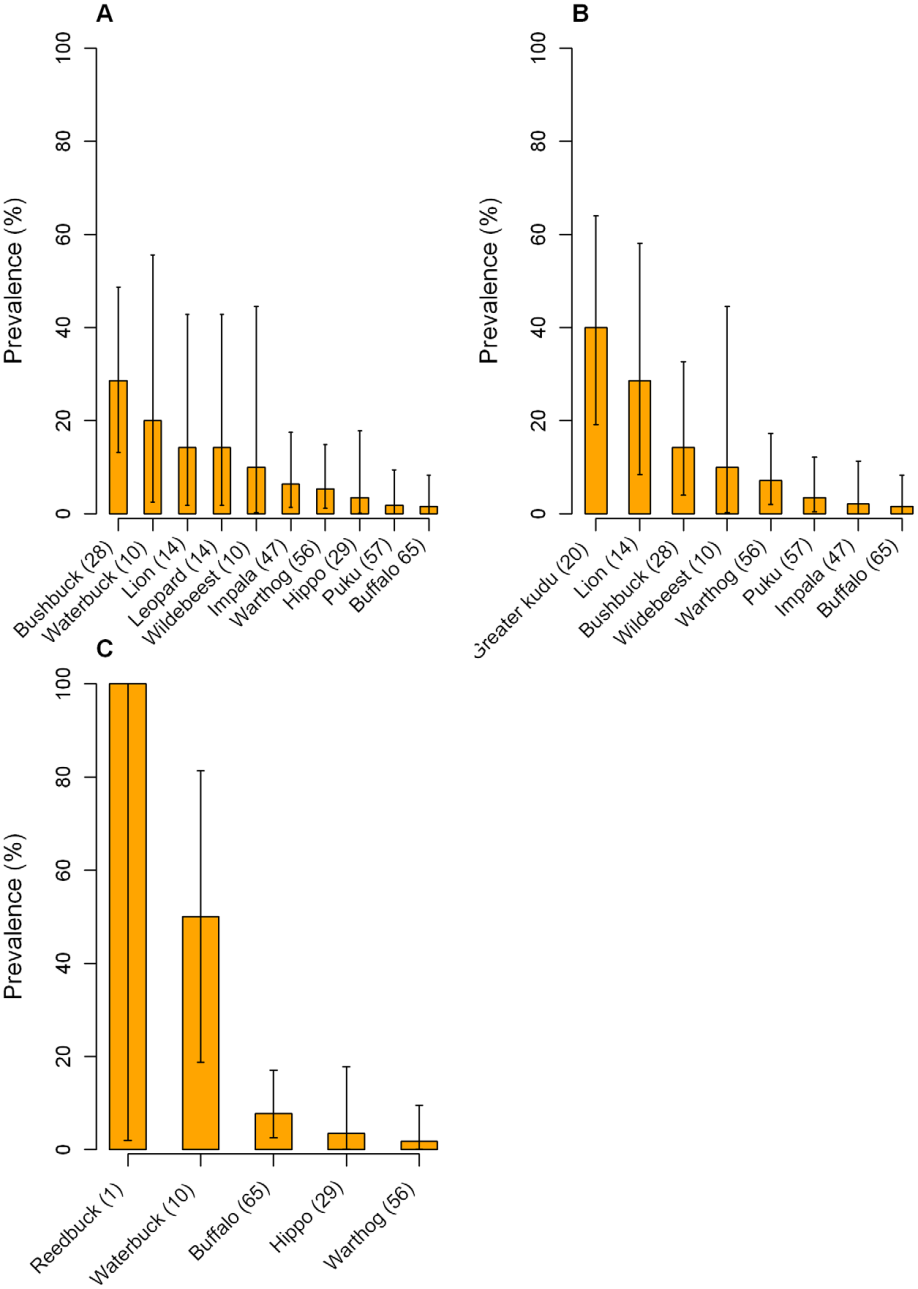
Bar charts of individual trypanosome species prevalence. Prevalence of (A) *T. brucei* s.l., (B) *T. congolense* and (C) *T. vivax* plotted against wildlife species. Only the species with at least one positive sample are shown in each graph. Error bars represent exact binomial 95% confidence intervals.

Host species was again significant as a factor (p = 0.042) and the bushbuck presented a significantly greater odds of being detected as infected (OR = 7.1, 95% CI: 1.7–29.33, p = 0.007). No other host species had a significantly greater likelihood of being detected as infected when compared with the reference warthog (Table 5). A bar chart of the prevalence detected in all species with at least one positive sample is presented in Figure 4 (A). Oversaturation of Whatman FTA cards also had a significant effect on *T. brucei* s.l. prevalence both when the complete dataset was analysed (p = 0.024) and when the samples collected by hunters were considered separately (p = 0.010). Over-saturated FTA cards were significantly less likely to be detected as positive with an OR of 0.4 (95% CI: 0.13–0.94, p = 0.038) using the complete dataset and 0.3 (95% CI: 0.11–0.81, p = 0.018) using the hunter dataset. Year also had a significant effect on the prevalence (p = 0.015), with samples collected in 2007 presenting a reduced likelihood of being detected as positive for *T. brucei* s.l. (OR = 0.2, 95% CI: 0.04–0.57, p = 0.005).

**Table 5:** Summary of the prevalence and odds ratios for *T. brucei* s.l., *T. congolense* and *T. vivax* in each host species.

Species (n)	*T. brucei* s.l. Prevalence % (95% CI)	OR(95% CI)	*T. congolense* Prevalence % (95% CI)	OR(95% CI)	*T. vivax* Prevalence % (95% CI)	OR(95% CI)
Warthog (56)	5.36 (1.12–14.87)	Referent	7.14 (1.98–17.29)	Referent	1.79 (0.05–9.55)	Referent
African painted dog (3)	0 (0.00–71.00)	N/A	0 (0.00–71.00)	N/A	0 (0.00–71.00)	N/A
Buffalo (65)	1.54 (0.04–8.28)	0.28(0.03–2.73)	1.54 (0.04–8.28)	0.20(0.02–1.87)	7.69 (2.54–17.05)	4.58(0.52–40.46)
Bushbuck (28)	28.57 (13.22–48.67)	7.07(1.70–29.33)**	14.29 (4.03–32.67)	2.17(0.50–9.40)	0 (0.00–12.36)	N/A
Crocodile (5)	0 (0.00–52.20)	N/A	0 (0.00–52.20)	N/A	0 (0.00–52.20)	N/A
Duiker (2)	0 (0.00–84.50)	N/A	0 (0.00–84.50)	N/A	0 (0.00–84.50)	N/A
Eland (1)	0 (0.00–98.00)	N/A	0 (0.00–98.00)	N/A	0 (0.00–98.00)	N/A
Elephant (7)	0 (0.00–41.00)	N/A	0 (0.00–41.00)	N/A	0 (0.00–41.00)	N/A
Giraffe (1)	0 (0.00–98.00)	N/A	0 (0.00–98.00)	N/A	0 (0.00–98.00)	N/A
Greater kudu (20)	0 (0.00–16.85)	N/A	40.00 (19.12–63.95)	8.67(2.24–33.58)**	0 (0.00–16.85)	N/A
Grysbok (4)	0 (0.00–60.25)	N/A	0 (0.00–62.50)	N/A	0 (0.00–60.25)	N/A
Hartebeest (4)	0 (0.00–60.25)	N/A	0 (0.00–62.50)	N/A	0 (0.00–60.25)	N/A
Hippo (29)	3.45 (0.09–17.76)	0.63(0.06–6.35)	0 (0.00–11.97)	N/A	3.45 (0.09–17.76)	1.96(0.12–32.59)
Hyaena (7)	0 (0.00–41.00)	N/A	0 (0.00–41.00)	N/A	0 (0.00–41.00)	N/A
Impala (47)	6.38 (1.34–17.54)	1.20(0.23–6.27)	2.13 (0.05–11.29)	0.28(0.03–2.62)	0 (0.00–7.55)	N/A
Leopard (14)	14.29 (1.78–42.81)	2.94(0.44–19.6)	0 (0.00–23.21)	N/A	0 (0.00–23.21)	N/A
Lion (14)	14.29 (1.78–42.81)	2.94(0.44–19.6)	28.57 (8.39–58.11)	5.20(1.11–24.31)*	0 (0.00–23.21)	N/A
Puku (57)	1.75 (0.04–9.39)	0.32(0.03–3.13)	3.51 (0.43–12.11)	0.47(0.08–2.69)	0 (0.00–6.28)	N/A
Reedbuck (1)	0 (0.00–98.00)	N/A	0 (0.00–98.00)	N/A	100.00 (2.00–100.00)	N/A
Roan (5)	0 (0.00–52.20)	N/A	0 (0.00–52.20)	N/A	0 (0.00–52.20)	N/A
Vervet monkey (1)	0 (0.00–98.00)	N/A	0 (0.00–98.00)	N/A	0 (0.00–98.00)	N/A
Waterbuck (10)	20.00 (2.52–55.61)	4.42(0.64–30.66)	0 (0.00–30.90)	N/A	50.00 (18.71–81.30)	55.00 (5.33–567.59)***
Wildebeest (10)	10.00 (0.25–44.50)	1.96(0.18–21.02)	10.00 (0.25–44.50)	1.44(0.14–14.45)	0 (0.00–30.90)	N/A
Zebra (27)	0 (0.00–12.78)	N/A	0 (0.00–12.78)	N/A	0 (0.00–12.78)	N/A
Total (418)	5.74 (3.71–8.42)	-	5.98 (3.91–8.70)	**-**	3.11 (1.67–5.26)	**-**

N/A – ORs could not be calculated due to zero prevalence or small sample size.

***p<0.001,

**p<0.01,

*p<0.05.

### Prevalence of *T. congolense*

The overall prevalence of *T. congolense* in all species was 6.0% (95% CI: 3.91–8.70%). Host species had a significant effect on the prevalence (p = 0.001) with greater kudu the species most likely to be detected as infected (OR = 8.7, 95% CI: 2.24–33.58, p = 0.002), followed by lion (OR = 5.2, 95% CI: 1.11–24.31, p = 0.036). No other species had a significantly increased risk of infection compared with the reference warthog species. A summary of the prevalence detected and OR for each species is shown in Table 5 and a bar chart of the prevalence detected for each species with at least one positive sample is shown in Figure 4 (B). No other factors had a significant effect on the *T. congolense* prevalence using the combined dataset. There was, however, a significantly lower likelihood of detecting *T. congolense* in the month of September (OR = 0.2, 95% CI: 0.05–0.64, p = 0.008).

### Prevalence of *T. vivax*

The *T. vivax* prevalence of 3.1% (95% CI: 1.67–5.26%) was lower than that for the two other trypanosome species that were investigated in this study. Host species had a significant effect on this prevalence (p = 0.002) and waterbuck was highly significant as a host with an OR of 55.0 (95% CI: 5.33–567.59, p = <0.001) (Table 5). Although buffalo also had an increased likelihood of being detected as infected, the OR was not significant. No other factors had significant effects on the *T. vivax* prevalence. Figure 4 (C) shows a bar chart of the prevalence of *T. vivax* in all wild animal species with at least one positive sample.

## Discussion

### Study design

The accurate diagnosis of trypanosomes in field surveys of wildlife populations has historically presented many challenges, in particular for *T. brucei* species. The protocol employed in this survey offered the advantage of an efficient method of sample collection and storage, combined with highly specific molecular techniques for diagnosis. The use of hunter kills as a source of surveillance material enabled a wide range of species to be sampled and increased the sample size obtainable from the resources available. Although the data generated was a convenience sample and is likely to be biased in terms of the sex and age distribution of the population sampled, this is a common problem with surveys of wildlife populations [19]–[21] and is difficult to overcome.

Where resources allow, molecular techniques of diagnosis offer the advantage over more traditional techniques of improved diagnostic specificity and sensitivity. This survey, along with a sister-project in Tanzania [37], represented the first use of the multispecies ITS-PCR [27] on field samples collected from free-ranging wildlife. A recent publication that used very similar protocols reported the specificity for *T. brucei* s.l. in a cattle population in Kenya to be 0.997 for the ITS-PCR and 0.998 for the TBR-PCR [38]. The sensitivities were not as high, however, with estimates of 0.640 and 0.760 respectively for the two techniques. The lower sensitivities achieved were attributed to the use of filter paper cards for DNA preservation and illustrate the main disadvantage of this technique of sample storage. The figures reported in this paper, therefore, although highly specific, are likely to underestimate the true prevalence of infection in wildlife. Only the data for *T. congolense* Forest, Kilifi and Savannah sub-species, *T. brucei* s.l. and *T. vivax* were used in the data analysis due to difficulties encountered in the accurate differentiation of bands at the sizes expected for *T. simiae* and *T. simiae* Tsavo. This limited the ability to detect mixed infections and the level reported (1.0%) might be lower than expected. However, it is possible that the high level of trypanosome challenge experienced by wild hosts in this ecosytem encourages the formation of a cross-immunity, as has been postulated for lions, and this may reduce the prevalence of mixed infections [39].

### Risk factors for infection

Host species was consistently identified as the most significant risk factor for infection with trypanosomes throughout the univariable analysis and no other factors had a significant effect after adjustment for confounding. The taxonomy grouping of species (p = 0.002) and habitat grouping (p<0.001) were also highly significant with blood meal preference grouping (p = 0.02) less so. When the residual deviances for each model were compared, the lowest value was obtained when no grouping was used at all (267.04) compared with the models containing the taxonomy grouping (304.03), habitat grouping (306.20) and blood meal preference grouping (328.57). As the residual deviance represents the unexplained deviance in the model, it is clear that much of the variation in infection rates occurs at the species level. However, it is also clear that complex interactions between parasite, host and vector determine the infection status of wildlife hosts.

At the individual trypanosome species level, host species was again the most significant factor explaining the variation in infection rates. Oversaturation of sample cards had a significant effect on *T. brucei* s.l. prevalence only and this suggests that the TBR-PCR may be more sensitive to inhibition by haem products than the multispecies ITS-PCR. The potential temporal effects detected in this study were most likely induced by the study design and resulted from sampling more species of low prevalence in the NLNP survey in 2007. It was difficult to investigate any potential confounding induced by the different sampling methods used as samples collected using the two methods were not collected from the same areas and species. This illustrates the difficulties with collecting data from wildlife suitable for a robust statistical analysis.

Although no association between the prevalence of trypanosome infection and age was evident in this study, the age distribution of the data limited the ability to investigate this as a risk factor. Very few studies have previously investigated the effect of age on trypanosome prevalence, but of those that have, one reported that the prevalence in buffalo peaked at two and a half years [8] and another found no statistically significant difference between the prevalence in young and old animals [17]. However, a more recent study of 13 lion prides in the Greater Serengeti Ecosystem in Tanzania where the actual age was known to within an accuracy of one month, reported that the prevalence of *T. brucei* s.l. infections showed a distinct peak and decrease with increasing age [39]. Most infections with *T. brucei* s.l. were cleared between three and five years of age and no human infective *T. b. rhodesiense* parasites were detected in lions over six years old. It was postulated that frequent challenge and an exposure dependent cross-immunity following infections with more genetically diverse species such as *T. congolense*, led to partial protection sufficient to prevent animals from harbouring human infective *T. b. rhodesiense*. As most samples in the study presented here were collected from older animals, it is possible that the prevalence in lions and potentially other species has been underestimated.

### The reservoir community

The results of this survey demonstrate the ability of trypanosomes to survive in a very wide variety of wildlife hosts. New identifications of *T. b. rhodesiense* in African buffalo and *T. brucei* s.l. in leopard (*Panthera* pardus) suggest that the reservoir community is even more diverse than previously thought. However, as illustrated by the species prevalence graphs, the majority of infections were concentrated in a smaller number of species. The majority of *T. brucei* s.l. infections were detected in four species, namely bushbuck, leopard, lion and waterbuck. Of these, the bushbuck was the only species to have a significantly greater likelihood of infection than warthog (OR: 7.1, 95% CI: 1.7–29.33). The bushbuck has previously been identified as an important reservoir host for *T. brucei* s.l. in the Luangwa Valley [1], [40] as well as in the Lambwe Valley in Kenya [3], [41]. Although overall densities of bushbuck are not high, they are locally abundant within the dense woodland and thicket vegetation common near the Luangwa River and its tributaries [42]. The high proportion of blood meals taken from this species by *Glossina pallidipes* tsetse [34], despite the relatively low overall population of bushbuck, suggests that there is a close ecological association between the two species. A transmission cycle involving this sedentary host and *G. pallidipes* is therefore likely to play an important role in maintaining foci of *T. brucei* in the Luangwa Valley, as has been proposed for the Lambwe Valley in Kenya [3].

The identification of *T. brucei* s.l. in samples from leopard in this study appears to be the first published record of infection in this species. Compared to many other protected areas in Africa, leopards are relatively common in the Luangwa Valley. It would appear that both lion and leopard are capable of supporting a moderate prevalence of *T. brucei* s.l. infection and there may be a secondary transmission cycle involving the carnivores. Interestingly it has been postulated that carnivores can become infected from their prey through abrasions in the oral mucosa and this has been demonstrated in artificial experiments [4]. Despite spending much of the day lying in dense thicket, these species also do not account for a high proportion of tsetse blood meals [34]. Trypanosome infections in these species may therefore be an example of bioaccumulation rather than vector transmitted disease, but no conclusions can be drawn on the route transmission from this study. However, considering their relatively low density, their contribution to trypanosome transmission is unlikely to be large whichever transmission route is involved.

The precise contribution of the waterbuck to the transmission of *T. brucei* s.l. is still unclear, although the high prevalence detected in this study and others [1], [7] suggests that they are highly susceptible to infection. Although they may be locally abundant overall densities are not high and they are rarely fed on by tsetse [34], [42]. They have been reported to produce allomones that repel tsetse and reduce the likelihood of feeding once landed [43]. However, they occupy a niche environment on the fringes of thicket and woodland and are clearly very susceptible to infection with all three trypanosome species. It has been postulated that their high susceptibility to infection has resulted from the fact that they are rarely challenged by infected tsetse bites [35], but the same can be said for many other species in which low infection rates are detected.

The diagnosis of *T. b. rhodesiense* in a sample from an African buffalo is, as far as the authors are aware, the first identification in this species. Buffalo are abundant in many savannah ecosystems and are capable of acting as a reservoir host for many pathogens of cattle, most probably because of their close phylogenetic relationship to the latter [44]. They have previously been demonstrated to be susceptible to sub-clinical infections with *T. brucei* s.l. [6], [8] so this finding is not surprising, but has important implications for the control of the disease. Buffalo are not sedentary animals and herds frequently move over large distances with the potential to disseminate infection to other host species. The finding of this parasite in Nyamaluma, not far from Mambwe and Msoro Districts where there have recently been large influxes of cattle and people, also raises concerns about the possibility of infection becoming established in the cattle population of the Luangwa Valley. In other parts of Africa, particularly Uganda, cattle have demonstrated to be effective maintenance hosts for *T. b. rhodesiense* [45], [46]. Areas with increasing populations of cattle adjacent to wildlife areas have also been identified as being at risk from epidemics of trypanosomiasis [47]. The prevalence of *T. brucei* s.l. in buffalo was relatively low, a finding that is in keeping with the moderate level of blood meals coming from this species despite their relative abundance [34], [42]. The only other positive identification of *T. b. rhodesiense* in this study was in a bushbuck in Musalangu GMA. This subspecies has previously been isolated from a bushbuck in the Luangwa Valley [23] as well as in the Lambwe Valley in Kenya [41] and its important role within the community of hosts for *T. brucei* s.l. has already been outlined.

The overall prevalence of *T. b. rhodesiense* detected in the study was relatively low at 0.5% (95% CI: 0.06–1.72%) suggesting that approximately 8% of all *T. brucei* s.l. identifications were *T. b. rhodesiense*. However, given that the majority of TBR-PCR positive samples did not test positive for PLC gene, it is likely to be an underestimate of the true prevalence. Even allowing for this, it is clear that the human infective subspecies is maintained at low levels by the reservoir community alongside a rich diversity of other trypanosome species. This might seem surprising given that wildlife hosts have long been regarded as the natural host for this parasite. It is in stark contrast to the ecological picture in Uganda where the human infective parasites circulate efficiently between cattle and man against a background of reduced biodiversity [48]. This raises the possibility that maintenance of biodiversity within the Luangwa Valley ecosystem has influenced the limited emergence of this parasite, although the data in this study are insufficient to prove this and many factors have been implicated in determining the patterns of parasite species richness [49], [50]. As spillover from wildlife has often been implicated as a risk factor for human infection [39], [51], the possibility that maintaining biodiversity might, conversely, limit the risk of infection in some situations warrants further investigation.

Of interest from a conservation perspective was the identification during the study of *T. brucei* s.l. in two black rhinoceros (*Diceros bicornis*) that had recently been re-introduced into the Luangwa Valley from a tsetse free area of South Africa (and were therefore not included in the data analysis). Histopathology of the brain from one rhinoceros which had died revealed severe meningo-encephalitis that was considered to be consistent with a diagnosis of clinical trypanosomiasis. Laboratory analysis of blood samples provided positive identification of *T. brucei* s.l. using the TBR-PCR and the GPI-PLC gene was positively identified using the SRA-PCR. Interestingly, the rhinoceros samples both had very strong positive GPI-PLC bands in comparison to other *T. brucei* s.l. positive samples where the GPI-PLC band was often negative, a finding which is suggestive of a higher level of parasitaemia in this species. The only clinical signs observed in the rhinoceros were depression and poor condition, although it was not examined by a veterinary surgeon. Although the final cause of death may be attributed to trypanosomiasis, it is not clear if it was a primary or secondary problem. Trypanosomiasis, including infection with *T. brucei* s.l., has been implicated previously in the post-translocation deaths of rhino [52]–[54].

The community of reservoir hosts for *T. congolense* would appear to be wider than that for the other trypanosome species, with members of the Bovidae family most frequently represented. Again, two separate transmission routes would appear to occur, one involving many of the ungulate species that are regularly fed on by tsetse and a second one involving the carnivores and possible oral transmission. Of the ungulates, a significant prevalence was detected in this study in greater kudu (OR = 8.7, 95% CI: 2.24–33.58), with moderate levels of infection in bushbuck and warthog. Both greater kudu and bushbuck are preferred hosts for *G. pallidipes* [34] and are sedentary hosts living largely in thicket or dense woodland, which is the prime habitat for this species of tsetse. This contrasts with the situation regarding warthog, where a close ecological association with *G. m. morsitans* has been described [34], [55]. A significant prevalence of infection was detected in lion in this study (OR = 5.2, 95% CI: 1.11–24.31), but, as with *T. brucei* s.l, it is doubtful that they form an important component of the community of reservoir hosts in terms of onwards disease transmission.

It is less straightforward to draw conclusions about the epidemiology of *T. vivax* infections in wildlife as the overall prevalence detected was much lower. Of all species sampled, waterbuck was the only species with a significant likelihood of infection (OR = 55.0, 95% CI: 5.33–567.59). Although this is clearly a significant odds ratio, the precise contribution of waterbuck to the transmission of infection is unclear and the reasons for this are as discussed for *T. brucei* s.l. A moderate prevalence with *T. vivax* was also detected in the more abundant buffalo, with occasional infections in other ungulates. Previous surveys have suggested that bushbuck and greater kudu are also capable of supporting *T. vivax* infections [7], [13] and agree with the high levels of infection detected in waterbuck [1], [7], [13]. Therefore, although the epidemiological picture is less clear for this species of trypanosome, it is likely that a transmission cycle involving the bovinae sub-family is the most important component of the reservoir.

### Epidemiology of trypanosomiasis

The Luangwa Valley ecosystem is unusual in modern day Africa due to the limited level of contact between domesticated livestock and wildlife. The results of the survey presented here along with historical surveys conducted in the valley [1], [7], [13] suggest that the epidemiology of trypanosomiasis has remained largely unchanged over the last century. This is in keeping with consistent land use patterns with an almost complete absence of livestock and only a modest change in the human population over the same time period. Infection rates in many species in this survey were comparable with previous surveys in the Luangwa Valley (Table 6). However, in recent years an influx of people and livestock into the Msoro and Mambwe Districts of central Luangwa Valley has led to the development of a new wildlife / livestock / human interface. An investigation into the prevalence of trypanosomiasis in domestic livestock at the site of this new interface in Msoro District, revealed infection rates of 33.3% in cattle, 20.9% in pigs, 27.6% in sheep and 10.2% in goats [56]. Although the laboratory protocol differed slightly from that used in this study, the same multispecies ITS-PCR was used. This is a much higher prevalence than that found in the surrounding wildlife population and represents a significant departure from the historical situation, with ramifications both for trypanosomiasis transmission and that of other infectious diseases. New interfaces have been identified as an important factor in disease transmission [19] and areas surrounding these interfaces have been identified as being at risk of epidemics of bovine trypanosomiasis [47]. Prevalence data produced from trypanosome surveys in neighbouring countries are not directly comparable due to the different diagnostic techniques used, but in general the prevalence recorded has been lower than that in the Luangwa Valley [57], [58].

**Table 6:** Summary of trypanosomes detected in previous surveys of wildlife in the Luangwa Valley by Kinghorn *et al* (1913), Keymer (1969) and Dillmann (1979).

*Species*	*Survey*	*Duttonella*	*Nannomonas*	*Trypanozoon*	*Mixed infections*	*Positive/Total (%)*	*Positive/Total (%)*
Buffalo	Keymer	-	-	-	-	0 / 4 (0)	2 / 23 (9)
	Dillmann	1	1	-	-	2 / 19 (11)	
Bushbuck	Kinghorn	-	4	2	-	6 / 9 (67)	22 / 38 (58)
	Keymer	1	1	-	1	3 / 6 (50)	
	Dillmann	3	7	1	2	13 / 23 (57)	
Civet	Dillmann	-	1	-	-	1 / 6 (17)	1 / 6 (17)
Duiker	Dillmann	1	2	-	-	3 / 7 (43)	3 / 7 (43)
Eland	Dillmann	-	1	-	1	2 / 3 (67)	2 / 3 (67)
Elephant	Kinghorn	1	-	-	-	0 / 1 (0)	2 / 21 (10)
	Dillmann	-	2	-	-	2 / 20 (10)	
Giraffe	Dillmann	-	-	1	-	1 / 1 (100)	1 / 1 (100)
Greater kudu	Kinghorn	-	4	-	-	4 / 7 (57)	15 / 21 (71)
	Keymer	-	-	-	1	1 / 1 (100)	
	Dillmann	3	3	-	4	10 / 13 (77)	
Hartebeest	Kinghorn	-	-	1	-	1 / 6 (17)	1 / 7 (14)
	Keymer	-	-	-	-	0 / 1 (0)	
Hippopotamus	Kinghorn	-	-	-	-	0 / 1 (0)	4 / 251 (2)
	Dillmann	-	-	4	-	4 / 250 (2)	
Hyaena	Dillmann	-	2	2	-	4 / 7 (57)	4 / 7 (57)
Impala	Kinghorn	-	-	1	1	2 / 29 (7)	3 / 59 (5)
	Keymer	-	-	-	-	0 / 7 (0)	
	Dillmann	-	1	-	-	1 / 23 (4)	
Lion	Kinghorn	-	-	-	-	0 / 2 (0)	6 / 8 (75)
	Dillmann	-	3	3	-	6 / 6 (100)	
Puku	Kinghorn	1	-	-	-	1 / 10 (10)	3 / 39 (8)
	Keymer	-	-	1	-	1 / 5 (20)	
	Dillmann	1	-	-	-	1 / 24 (4)	
Roan	Kinghorn	-	1	-	-	1 / 8 (13)	3 / 19 (16)
	Dillmann	-	2	-	-	2 / 11 (18)	
Warthog	Kinghorn	-	-	1	-	1 / 9 (11)	7 / 36 (19)
	Keymer	-	-	-	-	0 / 3 (0)	
	Dillmann	-	5*	1	-	6 / 24 (25)	
Waterbuck	Kinghorn	4	4	3	6	17 / 28 (61)	36 / 55 (65)
	Keymer	3	1	-	-	4 / 7 (57)	
	Dillmann	12	-	2	1	15 / 20 (75)	
Wildebeest	Kinghorn	-	-	-	-	0 / 2 (0)	1 / 10 (10)
	Keymer	-	-	-	-	0 / 3 (0)	
	Dillmann	1	-	-	-	1 / 5 (20)	
**Total**	**-**	**31**	**45**	**23**	**17**	**116 / 661 (17.5)**	

*Nannomonas infections were identified as *T. simiae*.

African wild dog, baboon, bat, bushpig, cane rat, crocodile, genet, grysbok, hare, jackal, leopard, mongoose, vervet monkey, porcupine, rhinoceros, serval, wild cat and zebra have all been sampled in the above surveys with negative results.

### Conclusions

*Trypanosoma* parasites circulate within a wide and diverse host community in this bio-diverse ecosystem. With the identification of the African buffalo and the leopard as new host species for *T. b. rhodesiense* and *T. brucei* s.l. respectively, it is clear that the reservoir community is wider than previously demonstrated. However, although the host range is very wide, the majority of infections are concentrated in a smaller number of species with a clear pattern of species forming the bulk of the reservoir community for each trypanosome species. Host species was the only consistent risk factor for infection identified in this study and, although many factors may interact to influence the trypanosome prevalence in wildlife, most of the variation in infection rates occurs at the species level. The epidemiology of trypanosomiasis in the Luangwa Valley has remained remarkably stable since the first survey in 1913, in keeping with consistent land use patterns despite some changes in the human population over that period. The recent influx of cattle and people from the plateau regions of Eastern Province represents a significant diversion from these land use patterns and will almost certainly result in changes in the epidemiology of trypanosomiasis in the Luangwa Valley, with cattle becoming increasingly important members of the reservoir community.
